# Survival analysis and prognostic factors of mature T-cell and natural killer/T-cell neoplasms in Colombian patients from 2008-2025

**DOI:** 10.3389/fonc.2026.1898102

**Published:** 2026-07-29

**Authors:** Juan F. Combariza-Vallejo, Rocío Orduz-Rodriguez, Natalia Gomez-Lopera, Claudia C. Colmenares-Mejia, Liliana Moreno-Martinez, Ana J. Salazar-Vargas, Marcela Godoy-Corredor, Mario Arturo Isaza-Ruget

**Affiliations:** 1 Clínica Colsanitas, Clínica Universitaria Colombia, Bogotá, Colombia; 2 Clínica Colsanitas, Laboratorio clínico y de patología, Bogotá, Colombia; 3 Fundación Universitaria Sanitas, Bogotá, Colombia

**Keywords:** lactate dehydrogenase (LDH), neutrophil to lymphocyte ratio (NLR), progression-free survival, survival analysis, T-cell lymphoma

## Abstract

**Background:**

T-cell lymphomas show global variation, but research in Latin America remains limited. The main outcome This study aimed to determine, subtype distribution frequency, treatment, and overall survival of T-cell lymphoma.

**Methods:**

We conducted a retrospective cohort study of 101 patients with mature T/NK-cell neoplasms (2008–2025). Survival (OS and PFS) was analyzed using Kaplan–Meier estimates and Cox models.

**Results:**

Median age was 58 years. PTCL-NOS (31.7%), anaplastic large cell lymphoma (20.8%), and extranodal NK/T-cell lymphoma (18.8%) were the most frequent subtypes. The 36-month OS was 57.8% and PFS was 42.1%. Significant prognostic heterogeneity was observed, with anaplastic large cell lymphoma showing the best and T-prolymphocytic leukemia the poorest outcomes. In multivariable analysis, ECOG status ≥2 independently predicted higher mortality (HR = 2.73, p<0.001), while low LDH levels reduced risk (HR: 0.22; 95% CI: 0.08–0.61; p = 0.003).

**Conclusions:**

These findings highlight the marked clinical heterogeneity of T-cell lymphomas and validate the prognostic value of ECOG and LDH in Latin America.

## Introduction

Mature T-cell lymphomas represent a rare and heterogeneous group of hematologic malignancies, comprising several distinct subgroups of non-Hodgkin lymphomas (NHLs) that are traditionally characterized by an aggressive clinical course and poor survival outcomes. These neoplasms arise from the dysregulation of signaling pathways crucial to normal T-cell development, differentiation, and virus-mediated oncogenesis (1). Unlike immature T-cell neoplasms, such as T-lymphoblastic leukemia/lymphoma, mature T-cell malignancies exhibit highly variable immunologic phenotypes, molecular profiles, genetic alterations, morphologies, and clinical features (2).

Globally, mature T-cell lymphomas are uncommon, accounting for approximately 10% to 15% of all NHL cases worldwide, and display striking geographic variation. The prevalence of T-cell lymphoma is slightly higher in Asia, accounting for 18%, followed by Central and South America (12.7%) than in the United States or Europe, where it is around 9.8% (3). Although these differences in geographic distribution are well recognized in the USA, Europe, and Asia, there is limited data from Colombia regarding epidemiology records of lymphoma. To the best of the authors’ knowledge, there is only one study in Colombia where it has been estimated that in a group of 819 patients with lymphomas, 4.4% corresponds to T-cell lymphoma (4).

The World Health Organization (WHO) classification of hemato-lymphoid tumors recognizes more than 30 definitive or provisional entities under mature T- and NK-cell neoplasms (5). The most common subtypes are PTCL not otherwise specified (NOS; 25.9%), Angioimmunoblastic type (18.5%), extranodal natural killer T-cell lymphoma, nasal type (NKTCL) (10.4%), and adult T-cell leukemia/lymphoma (ATLL; 9.6%) (6). Nevertheless, subtype prevalence varies substantially by region. In Central and South America, the most common subtypes were peripheral T-cell lymphoma not otherwise specified (PTC-NOS; 15-50%), followed by cutaneous T-cell lymphoma NOS (14- 42%) (7), whereas in East Asia, NKTCL is the most prevalent subtype (8).

These malignancies are typically diagnosed at an advanced stage in adult patients, they have an aggressive clinical course and an overall survival of three to five years (9). Compared to patients with B-cell lymphomas, patients with PTCLs exhibit highly aggressive clinical features, including B-symptoms, diffuse extranodal involvement, elevated serum beta-2 microglobulin and lactate dehydrogenase (LDH) levels, bulky disease, high Ki-67 proliferation indices, and p53 overexpression (10). A major challenge in PTCL management is the lack of highly effective frontline standards, which is currently provided by combination chemotherapy regimens involving CHOP (cyclophosphamide, doxorubicin, vincristine, and prednisone) or CHOP-like chemotherapy regimens. Brentuximab vedotin (which is a targeted therapy against the CD30 receptor), in combination with CHP (cyclophosphamide, doxorubicin, and prednisone), improves progression-free survival (PFS) and overall survival (OS) (11). First-line consolidation with high-dose therapy followed by autologous stem cell rescue (HDT/ASCR) may provide a survival benefit in high-risk patients with PTCL NOS and AITL (Angioimmunoblastic t-cell lymphoma) but not for other subtypes of PTCL (12).

Refractory or relapsed (R/R) disease after achieving remission with first-line therapy is common in peripheral T-cell lymphoma (PTCL), and its management poses a significant clinical challenge. The International Peripheral T-Cell Lymphoma Project reported a 5-year OS rate of less than 10% for R/R PTCL (13), Furthermore, the traditional salvage approach for Non-Hodgkin lymphomas using autologous transplantation has proven largely unsuccessful in this setting, yielding a median event-free survival (EFS) of only 5.3 months (14). Previous studies have demonstrated that PFS at 24 months (PFS24) is an essential milestone in stratifying patients with peripheral T-cell lymphoma (PTCL); survival beyond these time points is similar or equivalent to that of the general population (18).

Differences in the genetic background and environmental variables have been proposed to explain these geographical differences in subtype distribution and treatment response; however, the cause of these differences remains largely unexplained (15). To date, there is a lack of comprehensive studies examining subtype distribution, treatment response, and survival differences among T-cell lymphoma subtypes in Latin America, particularly in Colombia. Comparing epidemiology data from various regions of the world may provide new clues for future etiological investigations and treatments for this rare and heterogeneous disease. Accumulating evidence indicates that a high baseline neutrophil-to-lymphocyte ratio (NLR) correlates with poorer overall survival (OS; HR = 2.20, 95% CI: 1.71–2.83), without substantially affecting progression-free survival (PFS) (16). Consequently, we sought to investigate the role of the NLR as a prognostic marker for T-cell lymphomas in our cohort.

Given the limited information available in Colombia on this topic, we aimed to determine some demographic characteristics, subtype distribution frequency, treatment, and overall survival of T-cell lymphoma patients in our institution during the period 2008-2025.

## Materials and methods

This study followed STROBE recommendations for communicating results (17).

### Design

The study was conducted at Clínica Colsanitas, a Colombian hospital network with centers in Bogotá (Clínica Universitaria Colombia and Clínica Reina Sofía), Cali (Clínica Sebastián de Belalcázar), and Barranquilla (Clínica del Carmen and Clínica Iberoamérica). This institution is a reference center for hematological malignancies in Colombia and is also affiliated with an academic postgraduate institution (Unisanitas) in Bogotá.

The beginning of the follow-up was the moment of the hemato-oncological diagnosis, and the patients were followed until the last available follow-up medical appointment in the electronic medical record system.

### Patients

Inclusion criteria consisted of patients aged 18 years or older diagnosed *de novo* with T-cell lymphoma at Clínica Colsanitas. To ensure consistency, a hematopathologist reviewed all cases and classified them according to the latest WHO classification (2022).

We excluded patients with T-cell lymphoblastic lymphoma. We reviewed the hematology department’s database and selected patients who met the inclusion criteria.

### Outcomes

The main outcome was Overall Survival (OS), defined as the time in months elapsed from diagnosis until death from any cause. The secondary outcome was Progression Free Survival (PFS), defined as the time elapsed from the date of diagnosis until to the date of disease progression or death for any cause.

For the analysis proposal, we calculated the neutrophil-to-lymphocyte ratio (NLR) for each patient at the diagnostic time before starting any treatment with chemotherapy or steroids. The NLR test’s operative characteristic has been calculated for 2-year PFS. Subsequently, we divided patients into two groups, patients with higher levels of NLR were included in group one, while patients with lower levels in group two. Both groups were followed for at least 24 months from diagnosis.

### Variables

From electronic medical records we retrieved patient information, including demographic characteristics (age and sex), hematological diagnosis, B symptoms, initial site involvement (nodal or extranodal), disease stage according to the Ann Arbor classification (19), HIV comorbidity, treatment, relapse, complete response (CR) to therapy according to the International Working Group criteria 12 and death. Hematological diagnosis was classified as Peripheral T-cell lymphomas not otherwise specified (PTCL NOS), Angioimmunoblastic T-cell lymphoma (AITL), anaplastic large cell lymphoma (ALCL), Extranodal NK/T-cell lymphoma (ENKL), Large granular lymphocytic leukemia(LGL), T-cell prolymphocytic leukemia (T-PLL), adult T-cell leukemia/lymphoma (ATLL), and hepatosplenic T cell lymphoma (HSTL). Therapy approaches were classified as CHOEP, CHOP, PUVA and others that included CHOEP/MTX, Brentuximab, CVP, DEVIC, FCM, Phototherapy, Gemcitabine, GEMOX, ICE, Interferon, Methotrexate, Methotrexate/Cytarabine, MTX, Prednisone, R-DEVIC, radiotherapy only, RT DEVIC, SMILE.

### Statistical analysis

In the initial descriptive analysis for quantitative variables, continuous variables were presented as mean with standard deviation or median with interquartile range (IQR), for qualitative variables absolute frequencies and relative frequencies were measured. To evaluate association between nominal variables Chi square test or Fisher test was performed and for quantitative variables t student, and in case of different than normal distribution Wilcoxon test was performed.

We estimated OS and PFS using the non-parametric Kaplan-Meier method with confidence intervals of 95% and compared the probability of survival using the log-rank test. We used a proportional hazard regression (Cox) to evaluate factors influencing OS and PFS.

Once the data were collected, we used a mathematical formula dividing the absolute neutrophil count by the absolute lymphocyte count of each patient. A receiver operating characteristic (ROC) curve was constructed with the NLR variable and the outcome of interest (2 year PFS) to obtain sensitivity, specificity negative, and positive predictive value, and the optimal cutoff point to discriminate the population was determined. The sample was then divided accordingly for analysis. Because the primary outcome was PFS, a time-dependent event, we also calculated the time-dependent ROC curve and showed cumulative sensitivity and dynamic specificity for the performance evaluation of the NLR.

We considered biologically relevant factors and parameters with p value less than 0.20 in the bivariable test were included as covariates in the multivariable analysis. A p-value of less than 0.05 was considered statistically significant. All analyses were conducted using R version 4.4.1 (2024-06-14) -- “Race for Your Life” (20).

The Fundación Universitaria Sanitas (Unisanitas) institutional ethics committee approved the study.

## Results

### Population characteristics and subtype distribution

A total of 101 patients diagnosed with different subtypes of T-cell lymphoma were included. The median age at diagnosis was 58 years (IQR 43–65), and a balanced sex distribution was observed, with 51.5% male patients. The most frequent histological subtype was peripheral T-cell lymphoma not otherwise specified (PTCL-NOS), accounting for 31.7% of cases, followed by anaplastic T-cell lymphoma (20.8%) and extranodal NK/T-cell lymphoma, nasal type (18.8%). Complete cohort details are presented in **Table 1**.

**Table 1:** Demographic characteristics and histological distribution.

Characteristic	Total (n = 101)
Age, median (IQR)	58 (43–65)
Male sex, n (%)	52 (51.5%)
Histological subtype, n (%)	
PTCL-NOS	32 (31.7%)
Anaplastic T-cell lymphoma. - ALK Positive - ALK Negative - Unknown ALK Status	21 (20.8%) 4 (3.9%) 13 (12.8%) 4 (3.9%)
Extranodal NK/T-cell lymphoma, nasal type	19 (18.8%)
Angioimmunoblastic T-cell lymphoma	9 (8.9%)
T-cell large granular lymphocytic leukemia	9 (8.9%)
Adult T-cell leukemia/lymphoma associated with HTLV	6 (5.9%)
T-prolymphocytic leukemia	4 (3.9%)
Hepatosplenic T-cell lymphoma	1 (0.9%)

IQR, interquartile range; PTCL-NOS, peripheral T-cell lymphoma not otherwise specified; HTLV, human T-lymphotropic virus.

Baseline characteristics at diagnosis revealed the following distribution for Ann Arbor clinical stages: stage I (n = 4, 8.8%), stage II (n = 23, 25.2%), stage III (n = 15, 16.5%), and stage IV (n = 45, 49.5%). An Eastern Cooperative Oncology Group (ECOG) performance status of 0–1 was documented in 42 patients (63.7%), whereas a status of ≥ 2 was observed in 7 patients (14.3%). Extranodal involvement was present in 52 cases (77.6%), with documented sites including the stomach, liver, bone, breast, skin, lung, paranasal sinuses, and the central nervous system (CNS). Specifically, bone marrow and CNS involvement were reported in 31 (34.5%) and 3 (3.5%) patients, respectively. Additionally, B symptoms were present in 44 patients (64.7%). The median lactate dehydrogenase (LDH) level was 251 (IQR: 191–459), and the median leukocyte count at diagnosis was 7,150 (IQR: 5,200–10,890). Furthermore, the median Ki-67 index in the pathology specimens was 80% (IQR: 70–90%). These data are presented according to lymphoma subtype in **Table 2**.

**Table 2:** Baseline characteristics specified for each lymphoma subtype.

Histological subtype		Ann Arbor clinical stages	ECOG	Extranodal involvement	Bone marrow involvement	CNS involvement
	N	91	49	67	90	86
T-cell large granular lymphocytic leukemia	N = 9^*1*^	I-II 0 (0%)III-IV 5 (100%)	0-1 3 (100%)>2 0 (0%)	4 (100%)	8 (100%)	0 (0%)
T-prolymphocytic leukemia	N = 4	I-II 0 (0%)III-IV 4 (100%)	0-1 3 (100%) >2 0 (0%)	3 (100%)	4 (100%)	1 (25%)
Adult T-cell leukemia/lymphoma associated with HTLV	N = 6^*1*^	I-II 0 (0%) III-IV 6 (100%)	0-1 2 (50%)>2 2 (50%)	3 (100%)	2 (33%)	1 (17%)
Extranodal NK/T-cell lymphoma, nasal type	N = 19	I-II 10 (56%) III-IV 8 (44%)	0-1 5 (83%) >2 1 (17%)	15 (100%)	2 (13%)	0 (0%)
Anaplastic T-cell lymphoma.	N = 21^*1*^	I-II 9 (45%)III-IV 12 (55%)	0-1 4 (80%) >2 1 (20%)	10 (67%)	2 (13%)	0 (0%)
Angioimmunoblastic T-cell lymphoma	N = 9	I-II 2 (22%)III-IV 7 (78%)	0-1 7 (100%) >2 0 (0%)	3 (50%)	3 (38%)	0 (0%)
Hepatosplenic T-cell lymphoma	N = 1^*1*^	I-II 0 (0%)III-IV 1 (100%)	0-1 1 (100%) >2 0 (0%)	1 (100%)	1 (100%)	0 (0%)
PTCL-NOS	N = 32^*1*^	I-II 10 (36%)III-IV 18 (64%)	0-1 17 (85%)>2 3 (15%)	13 (65%)	9 (28%)	1 (3.2%)
p-value^*2*^			0.6	0.049	<0.001	0.2

*^1^*n (%).

*^2^*NA; Fisher’s exact test.

### Treatment

This study analyzes the first-line treatment regimens used for patients diagnosed with PTCL and their associated response rates. We had many different types of treatment as first line approach in the entire observed cohort. Specifically, it was found that the use of CHOP (n = 19, 31.1%) and CHOEP (n = 18, 29.5%), were the most employed treatment options. Remained patients were treated with low doses methotrexate (n=6 9.8%), RT- Devic (n=6, 9.8%), Smile (n=4, 6.5%), FCM (Fludarabine, cyclophosphamide, mitoxantrone) (n=3, 4.9%), GEMOX (Gemcitabine and oxaliplatin) (n=1, 1.6%), and brentuximab (n=5, 7.7%), one with brentuximab as monotherapy and 4 with brentuximab plus chemotherapy. A subset of patients (n = 19, 18.8%) underwent hematopoietic stem cell transplantation (HSCT) following first-line therapy. The majority received an autologous transplant (n = 15), while the remaining four patients underwent allogeneic transplantation. Regarding the indication, 17 patients received the transplant as first-line consolidation, whereas the remaining two underwent the procedure for relapsed disease.

There were variations in the first-line treatment according to the histological subtype, CHOP-like protocols were the mainly treatment in PTCL-NOS 71.8%, ALCL 91.6% and AITL 75%, and for ENKL the main treatment was Rt- Devic in 50% of the cases.

Regarding first-line treatment outcomes, 44 patients (50.5%) achieved a complete response (CR), while a partial response (PR) and progressive disease (PD) were observed in 12 (13.8%) and 19 (21.8%) patients, respectively. Early mortality prior to response evaluation occurred in 12 patients (13.8%). CR rates varied according to the treatment regimen: 12 patients (66.6%) for CHOEP, 11 (57.9%) for CHOP, 4 (66.6%) for RT-Devic, 2 (40.0%) for Brentuximab-based protocols, and 1 (33.3%) for FCM. Stratified by lymphoma subtype, anaplastic large cell lymphoma (ALCL) exhibited the highest CR rate at 16 patients (76.2%), the ALCL ALK + 3 (75%) and ALCL ALk – 10 (76.9%) followed by peripheral T-cell lymphoma not otherwise specified (PTCL-NOS) at 14 (43.7%) and extranodal NK/T-cell lymphoma (ENKL) at 7 (36.9%).

### Survival analysis and histology-based risk assessment

**Figure 1:**
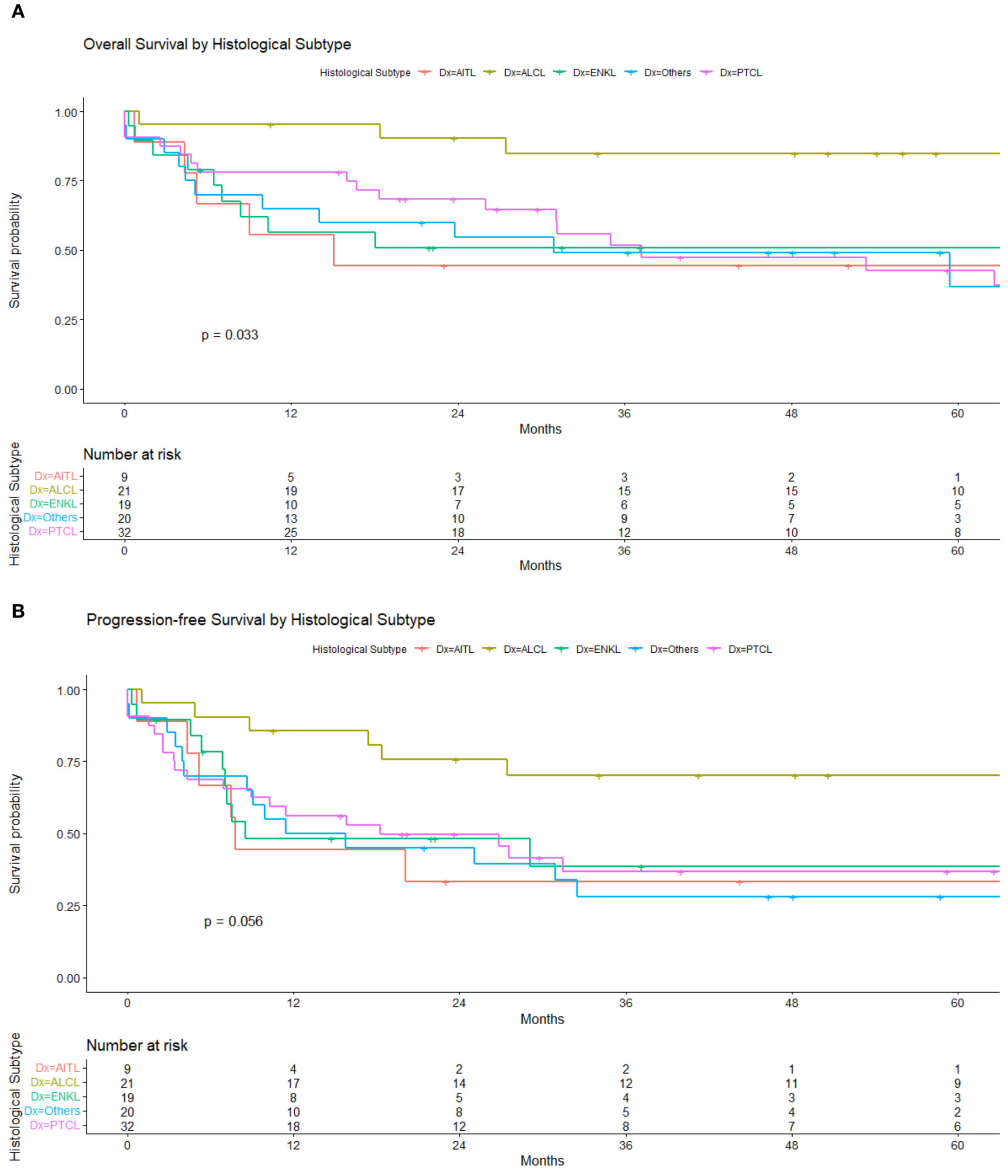
**(A)** Overall survival according to histological subtype. Peripheral T-cell lymphomas not otherwise specified (PTCL NOS), Angioimmunoblastic T-cell lymphoma (AITL), anaplastic large cell lymphoma (ALCL), Extranodal NK/T-cell lymphoma (ENKL), Others comprise Large granular lymphocytic leukemia(LGL), T-cell prolymphocytic leukemia (T-PLL), adult T-cell leukemia/lymphoma (ATLL), and hepatosplenic T cell lymphoma (HSTL). **(B)** Progression-free survival according to histological subtype. Peripheral T-cell lymphomas not otherwise specified (PTCL NOS), Angioimmunoblastic T-cell lymphoma (AITL), anaplastic large cell lymphoma (ALCL), Extranodal NK/T-cell lymphoma (ENKL), Others comprise Large granular lymphocytic leukemia(LGL), T-cell prolymphocytic leukemia (T-PLL), adult T-cell leukemia/lymphoma (ATLL), and hepatosplenic T cell lymphoma (HSTL).

The median follow-up time was 59 months (IQR: 31–92). Median overall survival (OS) for the entire cohort was not reached; the 36-month OS was 57.8% (95% CI: 48.4–69%). Likewise, median progression-free survival (PFS) was not reached, and the 36-month PFS was 42.1% (95% CI: 32.8–54%). Marked prognostic heterogeneity was observed among histological subtypes. Anaplastic T-cell lymphoma demonstrated the most favorable outcome, with a 36-month OS of 84.3% (69.4–100%), whereas T-prolymphocytic leukemia showed the poorest prognosis, with a 36-month OS of 25.0% (4.6–100%) and a median survival of 7.52 months. **Figure 1**.

### Prognostic factors

#### ROC curve analysis and cutoff performance for NLR and LDH

The median baseline NLR was 2.63 (IQR: 1.42–4.61). To evaluate its prognostic performance, a receiver operating characteristic (ROC) curve was constructed using 2-year PFS as the clinical outcome. At the optimal cutoff value of 3.59, the NLR achieved an area under the curve (AUC) of 0.68 (95% CI: 0.54–0.82), yielding a sensitivity of 79.6% and a specificity of 66.6%.

**Figure 2:**
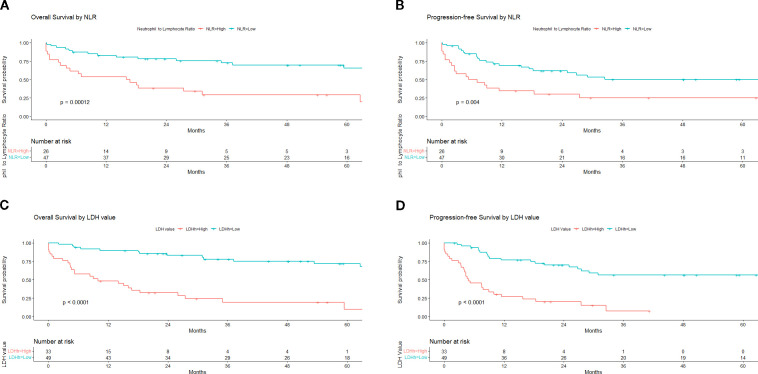
Overall survival and progression free survival according to NLR and LDH value. **(A)** Overall survival according to neutrophil to lymphocyte ratio. **(B)** Progression-free survival according to neutrophil to lymphocyte ratio. **(C)** Overall survival according to LDH value. **(D)** Progression-free survival according to LDH value.

Regarding lactate dehydrogenase (LDH), the median baseline level was 251 (IQR: 191–398). The corresponding ROC analysis for 2-year PFS identified an optimal LDH cutoff threshold of 273. This value demonstrated a superior predictive capacity, with an AUC of 0.78 (95% CI: 0.67–0.90), a sensitivity of 77.5%, and a specificity of 77.7%. **Figure 2**.

#### Survival analysis according to NLR and LDH value

##### Survival outcomes stratified by NLR

The median OS for the high NLR group was 6.18 months (95% CI: 2.57–NR) compared to 74.3 months (95% CI: 18.2–NR) for the low NLR group. The 36-month OS rates were 29.3% (95% CI: 15.8–54.3%) and 72.2% (95% CI: 60.5–87.4%) for the high and low NLR cohorts, respectively (HR = 0.28, 95% CI: 0.14–0.55; $p$ < 0.001). Similarly, the median progression-free survival (PFS) was 6.18 months (95% CI: 2.57–NR) in patients with high NLR versus 74.37 months (95% CI: 18.23–NR) in those with low NLR. The corresponding 36-month PFS rates were 25.2% (95% CI: 12.6–50.3%) and 50.2% (95% CI: 36.8–68.4%), respectively (HR = 0.41, 95% CI: 0.22–0.76; $p$ = 0.004) (**Table 3**).

##### Survival outcomes stratified by LDH

The median OS for the high LDH group was 9.93 months (95% CI: 4.57–26.0) compared to NR for the low LDH group. The 36-month OS rates were 19.4% (95% CI: 8.9–42.1%) and 77.9% (95% CI: 66.6–91.1%) for the high and low LDH cohorts, respectively (HR = 0.16, 95% CI: 0.08–0.32; $p$ < 0.001). Similarly, the median PFS was 4.87 months (95% CI: 3.93–11.5) in patients with high LDH versus NR in those with low LDH. The corresponding 36-month PFS rates were 7.58% (95% CI: 1.2–39.4%) and 56.4% (95% CI: 43.2–73.6%), respectively (HR = 0.21, 95% CI: 0.11–0.39; $p$ < 0.001).

### Univariate and multivariate analyses

In the univariate analysis, several factors were associated with inferior overall survival (OS). Specifically, Eastern Cooperative Oncology Group (ECOG) performance status ≥2 (hazard ratio [HR] 3.44), presence of B symptoms (HR 9.23), elevated lactate dehydrogenase (LDH) levels (HR 7.40), and bone marrow involvement (HR 1.89), and low neutrophil-to-lymphocyte ratio (NLR) (HR 0.24) were all statistically significant (p < 0.05).

To evaluate the independent effects of these variables, a multivariable Cox regression model was constructed, adjusted for ECOG performance status, LDH levels, and NLR. The final model (**Table 3**) demonstrated strong predictive performance, with a concordance index of 0.861. ECOG performance status remained the strongest independent prognostic factor [HR] 2.73; 95% CI: 1.66–4.51; p < 0.001). Likewise, LDH levels retained prognostic significance, with low LDH levels associated with a 77% reduction in mortality risk (HR 0.22; 95% CI: 0.08–0.61; p = 0.003). Although significant in the univariate analysis, NLR did not retain statistical significance in the adjusted model (p = 0.114) (**Table 4**).

**Table 3:** Overall survival, progression-free survival, and relative mortality risk according to histological subtype.

T-cell lymphoma classification	Median OS (95% CI)	36-month OS (95% CI)	Median PFS (95% CI)	36-month PFS (95% CI)	HR (95% CI)	p value
Peripheral T-cell lymphoma, NOS	37.2 (26–NR)	50.4% (34.6–73.5%)	18.2 (8.9–NR)	37.9% (23.2–62%)	5.42 (0.7–40)	0.10
Anaplastic T-cell lymphoma	NR	84.3% (69.4–100%)	NR	69.2% (51.3–93.4%)	1.2 (0.12–11.3)	0.88
Extranodal NK/T-cell lymphoma, nasal type	NR	52.4% (33–83.2%)	8.53 (7.1–NR)	38.3% (19.8–74.2%)	5.2 (0.6–41)	0.12
Angioimmunoblastic T-cell lymphoma	15.1 (5.2–NR)	41.7% (18.5–94%)	7.8 (5.2–NR)	16.4%	7.4 (0.86–63)	0.06
T-cell large granular lymphocytic leukemia	NR	88.9% (70.6–100%)	NR	50.8% (25.7–100%)	Reference	--
Adult T-cell leukemia/lymphoma associated with HTLV	31.3 (13.3–NR)	50% (5.1–100%)	17.7 (7.1–NR)	25% (5.1–100%)	10.5 (1.2–94)	0.03
T-prolymphocytic leukemia	7.52 (4.4–NR)	25% (4.6–100%)	7.1 (3.5–NR)	25% (4.6–100%)	16.7 (1.9–150.9)	0.01
Hepatosplenic T-cell lymphoma	23.8	0%	8.67	0%	10.8 (0.67–174)	0.09

OS, overall survival; PFS, progression-free survival; HR, hazard ratio; CI, confidence interval; PTCL-NOS, peripheral T-cell lymphoma not otherwise specified; HTLV, human T-lymphotropic virus; NA, not reached/not available.

**Table 4:** Univariate and multivariate Cox regression analyses of prognostic factors for overall survival.

Variable	Univariate analysis HR (95% CI)	P value	Multivariate analysis HR (95% CI)	P value
ECOG (≥2)	3.44 (1.81–6.55)	<0.001	2.73 (1.66–4.51)	<0.001
LDH (Low vs High)	7.40 (Reference)*	<0.001	0.22 (0.08–0.61)	0.003
NLR (Low)	0.24 (0.11–0.51)	<0.001	0.49 (0.20–1.18)	0.114
B symptoms	9.23 (2.19–38.8)	<0.001	--	--
Bone marrow involvement	1.89 (1.02–3.50)	0.041	--	--

*HR, hazard ratio; CI, confidence interval; ECOG, Eastern Cooperative Oncology Group performance status; LDH, lactate dehydrogenase; NLR, neutrophil-to-lymphocyte ratio; OS, overall survival.

## Discussion

Our study demonstrates the distribution of T-cell lymphoma subtypes in our population, characterized by marked prognostic heterogeneity among histological subtypes. PTCL-NOS was the most frequent subtype, whereas anaplastic T-cell lymphoma had the best overall survival, and T-prolymphocytic leukemia had the poorest clinical outcome. The 36-month OS was 57.8% (95% CI: 48.4–69%). and the 36-month PFS was 42.1% (95% CI: 32.8–54%). In addition, ECOG ≥2 and elevated LDH levels emerged as the main independent prognostic factors associated with inferior overall survival. These findings confirm the biologically diverse and clinically aggressive nature of T-cell lymphomas in our population.

Epidemiological studies indicate that the distribution of lymphoma subtypes varies significantly across geographic regions, which is attributed to a combination of genetic, infectious, and environmental factors specific to each region. In this report, we present the largest descriptive study conducted at a single institution in Colombia evaluating the distribution of T-cell lymphoma subtypes. Our findings indicate that T-cell lymphomas are uncommon in our country, representing 6.8% of all non-Hodgkin lymphomas, which falls within the previously reported range in Western countries, between 5% and 10% (21, 22). However, these results differ from those observed in Asian countries and other developing nations, where a higher proportion, ranging from 13% to 20% of all non-Hodgkin lymphoma cases, corresponds to T-cell or NK-cell lymphomas (23).

The distribution of T-cell lymphoma subtypes in our population may differ from that reported in the literature. The most frequent subtype in our cohort was PTCL-NOS, followed by anaplastic T-cell lymphoma. These subtypes are also prevalent in North America and Europe, where PTCL-NOS is the most common subtype, with a median age at diagnosis of 65 years (8). PTCL-NOS comprises a diverse group of complex disorders that cannot be classified into existing subtypes based solely on morphological criteria. The high number of PTCL-NOS cases may be attributed to difficulties in establishing specific lymphoma diagnoses, as previously reported in other Latin American countries (24).

Moreover, our population had a higher incidence of cutaneous and NK/T-cell lymphomas than in Western countries. This trend has also been observed in Asia and other Latin American nations, where these lymphoma types account for 7% to 10% of all non-Hodgkin lymphoma cases (22, 25). Extranodal NK/T-cell lymphoma is strongly associated with Epstein–Barr virus (EBV) infection, and genetic factors are believed to play an important role in its development (26). This type of lymphoma has shown a notable increase among indigenous populations in Guatemala, Chile, and Peru, possibly related to an Amerindian genetic component within these populations (27, 28). Several studies suggest that shared genetic characteristics between Asians and Native Americans originated from early migrations from Asia to the Americas through the Bering Strait, followed by long periods of isolation and diversification within the American continent (29). Genetic studies indicate that the Colombian population exhibits a genetic admixture composed of approximately 60–70% European ancestry, 20–30% Amerindian ancestry, and 10–20% African ancestry (30). The Amerindian ancestry present in our population may partially explain the observed findings.

In our cohort, the complete response rate was 50.0% alongside an early mortality rate of 13.8%. These outcomes directly influence both overall survival and the feasibility of subsequent consolidation strategies, such as autologous or allogeneic stem cell transplantation. This unfavorable scenario may be attributed to diagnostic delays stemming from inherent clinical challenges or systemic barriers to healthcare access. Such factors routinely culminate in clinical and functional deterioration, ultimately compromising the patients’ capacity to tolerate intensive first-line chemotherapy regimens. Furthermore, targeted therapies with more favorable toxicity profiles, such as brentuximab vedotin, have only recently gained regulatory approval in Colombia for the treatment of T-cell lymphomas. Consequently, the vast majority of patients in our cohort lacked access to these novel agents during the study period. Although the complete response (CR) rate in our cohort may appear low compared to modern frontline protocols—such as brentuximab combined with chemotherapy, which yields CR rates between 79% and 92% (31, 32), it does not differ significantly from outcomes observed with anthracycline-based regimens alone. Historically, conventional protocols like CHOP or CHOEP show CR rates ranging from 31% to 69% (33, 34).

The 36-month OS was 58.7%, although significant differences were observed according to T-cell lymphoma subtype. Overall, our findings are consistent with those reported by other large collaborative groups, such as the International T-Cell Lymphoma Project and the Latin American Lymphoproliferative Study Group (6, 25), which have reported overall survival values ranging from 22 to 49 months. Regarding subtype-specific outcomes, we observed extreme prognostic disparities. While anaplastic T-cell lymphoma demonstrated a favorable 36-month overall survival of 84.3%, subtypes such as T-prolymphocytic leukemia and HTLV-associated leukemia/lymphoma showed a significantly increased risk of death (HR 16.7 and 10.5, respectively). This finding is consistent with current evidence describing T-PLL as one of the most aggressive T-cell lymphoid neoplasms, characterized by rapid proliferation, resistance to conventional chemotherapy, and a median survival of less than 1 year in the absence of consolidation with allogeneic transplantation (35). Similarly, HTLV-associated leukemia/lymphoma is consistent with studies reporting particularly unfavorable outcomes related to severe immunosuppression, hypercalcemia, high tumor burden, and limited efficacy of conventional therapies (35, 36).

One of the most relevant findings of our study was the identification of ECOG performance status ≥2 as the strongest independent predictor of mortality. This result has important biological and clinical plausibility, as functional impairment simultaneously reflects tumor burden, systemic inflammatory status, and the patient’s physiological reserve. In fact, ECOG constitutes one of the core components of the main prognostic indices used in PTCL, including the International Prognostic Index (IPI), the Prognostic Index for T-cell Lymphoma (PIT), and the modified PIT (37). Our findings support the applicability of these prognostic tools in Latin American populations and reinforce the relevance of functional status as a universally reproducible clinical biomarker.

Likewise, elevated LDH retained prognostic significance in the multivariable model. LDH has consistently been recognized as an indirect marker of high cellular proliferation, accelerated tumor metabolism, and biological aggressiveness in aggressive lymphomas (38). In our cohort, low LDH levels were associated with a 77% reduction in the risk of death, confirming its utility as an accessible and low-cost prognostic biomarker. The persistence of LDH and ECOG as independent variables suggests that both factors capture complementary dimensions of tumor aggressiveness and clinical vulnerability.

The NLR showed a significant association in the univariate analysis, but this association lost significance after multivariable adjustment. Recent evidence has demonstrated that elevated NLR is associated with inferior OS and PFS in PTCL, likely reflecting systemic inflammation, tumor-related immunosuppression, and adaptive immune dysfunction. A meta-analysis published in 2021 (16) confirmed the adverse prognostic value of elevated NLR in PTCL, particularly in aggressive subtypes. However, the loss of significance in our model may be due to collinearity with clinical variables of greater prognostic weight, particularly ECOG and LDH, or to limitations in sample size.

The presence of B symptoms and bone marrow involvement were also associated with inferior OS in the univariate analysis, findings consistent with the literature. Both parameters reflect advanced systemic disease and higher tumor burden, factors historically associated with poorer therapeutic response and shorter survival (38). Nevertheless, these variables lost significance in the adjusted model, possibly due to biological overlap with other clinical markers included in the analysis.

Our study has several limitations, some of which are common to retrospective observational studies. Primarily, the missing data for some variables are attributable to the limited information available in medical records. Second, some histological subtypes were represented by only a few patients, limiting the precision of the estimates and contributing to the wide confidence intervals observed. Third, molecular data, ALK status, and detailed information on therapeutic strategies were unavailable, factors that are likely to have a significant influence on contemporary clinical outcomes. Finally, the therapeutic heterogeneity inherent to a real-world study may have impacted the observed survival outcomes.

Despite these limitations, our study provides important information for Latin American populations, given the scarcity of epidemiological studies on these diseases in the region. Further research is needed to better understand the biology of PTCL, to evaluate the roles of genetic and environmental factors in the development of T-cell lymphomas, and to deepen our understanding of disease etiology. This information will enable earlier detection and diagnosis, as well as more standardized treatment strategies, ultimately improving clinical outcomes for patients with T-cell lymphomas. Understanding these epidemiological patterns may also facilitate the development of therapeutic alternatives to improve cure rates and reduce late treatment-related adverse effects, thereby enhancing the quality of life of Colombian patients with T-cell lymphomas.

## Data Availability

The raw data supporting the conclusions of this article will be made available by the authors, without undue reservation.
